# *APOE*-associated lipid-handling macrophages in hepatocellular carcinoma: ligand–receptor communication and host *Apoe*-linked myeloid remodeling

**DOI:** 10.3389/fimmu.2026.1902143

**Published:** 2026-08-11

**Authors:** Tong Wu, Guijie Xin, Jie Zhou, Xiaomei Wang, Junqi Niu

**Affiliations:** 1 Department of Hepatology, Center of Infectious Diseases and Pathogen Biology, The First Hospital of Jilin University, Changchun, China; 2 Jilin Provincial Key Laboratory of Metabolic Liver Diseases, The First Hospital of Jilin University, Jilin University, Changchun, China; 3 China–Singapore Belt and Road Joint Laboratory on Liver Disease Research, The First Hospital of Jilin University, Jilin University, Changchun, China

**Keywords:** APOE, hepatocellular carcinoma, lipid metabolism, tumor microenvironment, tumor-associated macrophages

## Abstract

**Background:**

Hepatocellular carcinoma (HCC) arises in the liver, an organ with active lipid metabolism, and is accompanied by marked myeloid-cell remodeling. Whether lipid-handling macrophage states are enriched in HCC and how they relate to candidate ligand–receptor communication and tumor microenvironmental remodeling remain unclear.

**Methods:**

We analyzed paired tumor and adjacent liver single-cell transcriptomic data from 10 HCC patients. Tumor-associated lipid-handling candidates were screened using immune-cell composition, metabolic pathway activity, and tumor-upregulated lipid-related gene programs, with *APOE* prioritized for downstream analysis. Myeloid reclustering, module scoring, and CellChat analysis were used to characterize *APOE*-positive macrophages and infer *APOE*-positive macrophage-centered candidate communication. Hepa1–6 subcutaneous tumor models, an *Nras-Myc*-driven liver tumor model, mouse single-cell transcriptomics, and bone marrow-derived macrophage lipid-loading experiments were used to assess host *Apoe* deficiency, tumor-cell *Apoe* perturbation, and macrophage neutral lipid accumulation.

**Results:**

HCC tumors showed altered immune-cell composition and immune-metabolic pathway activity compared with adjacent liver tissues. Among tumor-upregulated lipid-related candidates, *APOE* was prioritized because of its tumor-associated expression, consistent ranking across lipid-related screening strategies, and functional relevance to lipoprotein and cholesterol handling. *APOE*-positive macrophages were enriched in tumors and displayed lipid-routing, cholesterol-handling, endolysosomal, and redox-adaptive programs. CellChat analysis identified candidate ligand–receptor interactions involving *APOE*-positive macrophages, including an *APOE*–*TREM2*-related incoming interaction and *SPP1*–integrin/*CD44* outgoing interactions. In the immunocompetent Hepa1–6 model, host *Apoe* deficiency attenuated tumor growth and was accompanied by reduced vascularization and increased CD8-positive cell infiltration, whereas tumor-cell *Apoe* knockdown did not significantly affect tumor volume or tumor weight in immunodeficient mice. In the *Nras-Myc* liver tumor model, *Apoe* deficiency reduced tumor burden and proliferative activity and was accompanied by a shift in tumor macrophage and monocyte programs away from lipid-processing and phagolysosomal activity toward inflammatory chemokine-associated activity. In lipid-loaded bone marrow-derived macrophages, *Apoe* deficiency increased neutral lipid accumulation.

**Conclusion:**

This study links *APOE*-associated macrophage lipid handling to inferred ligand–receptor communication and host *Apoe*-linked microenvironmental remodeling in HCC. These findings highlight an *APOE*-linked myeloid lipid-handling program as a potential component of immune microenvironmental remodeling during HCC development.

## Introduction

Primary liver cancer remains a major global cancer burden. According to GLOBOCAN 2022, there were approximately 866,000 new cases and 759,000 deaths from liver cancer worldwide, making it one of the leading causes of cancer-related mortality (1). Hepatocellular carcinoma (HCC) accounts for the vast majority of primary liver cancers. Its development is usually not an isolated malignant transformation event, but the result of long-standing chronic liver injury, inflammation, fibrosis, vascular remodeling, and metabolic dysfunction (2, 3). This metabolic dimension aligns with broader evidence that metabolic reprogramming can support malignant progression across tumor contexts (4, 5). In recent years, immune checkpoint inhibitor-based regimens, represented by atezolizumab plus bevacizumab, have improved the systemic treatment landscape for unresectable HCC (6). However, durable clinical benefit remains limited to a subset of patients, and a substantial proportion of patients show primary non-response or subsequent disease progression. These observations suggest that the efficacy of immunotherapy in HCC is not determined solely by checkpoint pathways, but is also shaped by tumor metabolic states, myeloid-cell remodeling, and local immunosuppressive programs (7, 8).

The immune microenvironment of HCC has distinct organ-specific features. The liver contributes to peripheral immune tolerance and also performs core metabolic functions, including lipoprotein assembly, cholesterol transport, fatty acid oxidation, and detoxification metabolism (3, 8). In this context, the liver’s active lipid metabolic environment may shape immune-cell functional states and should be considered when analyzing the HCC immune microenvironment. After tumor development, malignant cells and the surrounding stroma can continuously alter local nutrient availability, lipid and cholesterol metabolism, redox pressure, and the demand for clearance of dying cells. These changes affect not only tumor cells themselves, but also the functional states of infiltrating immune cells (8, 9). Among these cells, macrophages are of particular interest. Tumor-associated macrophages (TAMs) have phagocytic, lipid-handling, tissue-repair, and immune-regulatory functions, and can participate in shaping antitumor immunity by influencing inflammation, T-cell infiltration, angiogenesis, and extracellular matrix remodeling (10, 11). Previous single-cell studies in HCC have shown that tumor-infiltrating myeloid cells are not a uniform immunosuppressive population, but instead comprise multiple states related to inflammation, tissue residency, lipid handling, antigen presentation, and tumor-promoting functions (12–15). Therefore, a simple M1/M2 framework is no longer sufficient to explain the functional heterogeneity of TAMs in HCC (16). Beyond the tumor setting, spatially and transcriptionally distinct monocyte-derived macrophage populations have also been identified in injured mouse livers, including necrotic lesion-associated states implicated in dead-cell debris clearance and lesion resolution (17).

Lipid-associated macrophages and *TREM2*-positive macrophages have been reported to participate in metabolic liver disease, tissue repair, and remodeling of the tumor microenvironment, and they can show different functions across disease stages and tissue contexts (18–20). For example, during repair in metabolic dysfunction-associated steatohepatitis (MASH), *TREM2*-positive lipid-associated macrophages can contribute to apoptotic-cell clearance, inflammatory resolution, and tissue repair (18, 19). By contrast, recent work in HCC suggests that tumor cells with specific metabolic features can induce protumor *TREM2*-positive TAM states through oxidized low-density lipoprotein (oxLDL)-related metabolic crosstalk and promote immunosuppression (20). These findings suggest that lipid sensing and lipid handling may participate in the remodeling of myeloid-cell states in HCC. However, which lipid-handling programs contribute to tumor-enriched TAM states in HCC, how these states are connected to tumor-associated cell–cell communication, and whether they participate in supporting tumor growth remain incompletely understood.

To address these questions, we analyzed single-cell transcriptomic data from paired tumor and adjacent liver tissues from 10 patients with HCC, focusing on lipid-related immune programs that may connect tumor-associated immune remodeling with macrophage-centered ligand–receptor communication within the tumor microenvironment. We screened lipid-handling candidate factors from three complementary perspectives: immune-cell composition, metabolic pathway activity, and tumor-upregulated lipid-related genes. *APOE* was prioritized for further analysis because of its tumor-associated expression, association with myeloid/macrophage states, consistency across lipid-related screening strategies, and established role in lipoprotein transport and cholesterol redistribution (21). Genetic studies have also suggested that the *APOE* locus is associated with HCC susceptibility (22, 23). We then characterized the enrichment, lipid-handling features, and inferred ligand–receptor communication patterns of *APOE*-positive macrophages in HCC tumors. Finally, we used Hepa1–6 subcutaneous tumor models, an *Nras-Myc*-driven liver tumor model, and mouse single-cell transcriptomic analysis to assess the relationship between *Apoe* perturbation, tumor growth, and host microenvironmental remodeling *in vivo*.

## Materials and methods

### Single-cell RNA sequencing data processing

The single-cell RNA-seq dataset HRA001748 was obtained from the Genome Sequence Archive for Human (15). The dataset contained primary HCC tumors and paired tumor-adjacent liver tissues from 10 patients. Cells were retained if they had a mitochondrial transcript percentage <25%, a hemoglobin transcript percentage <5%, a ribosomal transcript percentage >5%, and at least 500 detected genes. Genes detected in fewer than three cells were removed. Ambient RNA contamination was estimated with DecontX, and cells with contamination scores ≥0.50 were excluded (24).

The retained cells were normalized and processed in Seurat (25). Highly variable genes were selected using the vst method, and cell-cycle-related genes were removed from the highly variable gene set before scaling, principal component analysis, and downstream clustering. Harmony was used for batch correction by sample identity, followed by nearest-neighbor graph construction, clustering, and uniform manifold approximation and projection (UMAP) visualization (26). Low-quality or doublet-like clusters were removed after manual inspection. Final clusters were annotated using canonical lineage markers and published HCC single-cell atlases (12–15).

### Metabolic analysis and lipid-related candidate selection

Metabolic pathway activity was scored in major immune-cell populations using scMetabolism with Kyoto Encyclopedia of Genes and Genomes (KEGG) pathways and AUCell (27–29). Pathway scores were summarized by patient, sample, and cell type; sample-cell-type combinations with fewer than 30 cells were excluded. For each pathway, sample-level mean scores were z-scored across samples, and tumor-minus-adjacent z-score differences were calculated to summarize tumor-associated metabolic changes.

For gene-level screening, tumor-versus-adjacent statistics were calculated within each major immune-cell type using sample-level aggregated expression. Paired Wilcoxon signed-rank tests were used when paired samples were available and unpaired Wilcoxon tests otherwise. *P* values were adjusted using the Benjamini–Hochberg method. Genes were retained if they were upregulated in tumors across all seven immune-cell groups and reached nominal significance (*P < 0.05*) in at least six groups. The retained genes were evaluated using lipid-related Gene Ontology (GO) biological process terms, Reactome/KEGG pathways, and a focused lipid-related gene filter, then prioritized by summed tumor-upregulated log fold change and consistency across lipid-related filtering strategies (28, 30, 31).

### Myeloid reclustering and module scoring

Myeloid cells were extracted from the global atlas and reprocessed using the same single-cell workflow. Mixed-lineage or low-quality clusters were removed after manual inspection. The remaining cells were annotated using canonical myeloid markers, subtype-specific genes, and published HCC myeloid single-cell studies (12–15). *APOE*-positive macrophages were defined as an *APOE*-enriched macrophage state identified by unsupervised myeloid reclustering and marker-based annotation. Myeloid subtype proportions were calculated as percentages of total myeloid cells and compared between paired tumor and adjacent samples.

Myeloid functional states were assessed using literature-informed curated gene modules representing macrophage-state, antigen-presentation, lipid/cholesterol-handling, endolysosomal, and redox-related programs. For each module, detected genes were scaled within the relevant cell population, and mean scaled expression was used as the cell-level module score. Scores were summarized by myeloid subtype or at the patient-sample level. For comparisons involving *APOE*-positive macrophages, samples with fewer than five cells assigned to this annotated macrophage state were excluded. Composite *APOE/*lipid-related or lipid-handling scores were calculated as the mean of the corresponding standardized module scores.

### Cell–cell communication analysis

Cell–cell communication was inferred with CellChat using separate tumor and adjacent-tissue objects (32). Cell groups were defined from the global cell-type annotation and refined myeloid annotation, with the annotated *APOE*-positive macrophage state analyzed as an independent group. Groups were downsampled to a maximum of 500 cells, and groups with fewer than 10 cells were excluded.

Communication probabilities were calculated from normalized RNA expression with population-size correction and 50 bootstrap iterations. CellChat-inferred candidate ligand–receptor interactions involving *APOE*-positive macrophages as senders or receivers were extracted and compared between tumor and adjacent tissues. The selected *APOE*-positive macrophage-centered ligand–receptor pairs are provided in **Supplementary Table 5**.

### Generation of *Apoe*-knockdown Hepa1–6 cells

Mouse hepatoma Hepa1–6 cells were purchased from Shanghai Fuheng Biotechnology Co., Ltd. (Shanghai, China) and cultured in DMEM supplemented with 10% fetal bovine serum at 37 °C in a humidified incubator containing 5% CO_2_. Cells were tested for mycoplasma contamination and confirmed to be mycoplasma-free before use. Stable *Apoe*-knockdown Hepa1–6 cells were generated using lentiviral shRNA. The *Apoe*-targeting shRNA vector pLV3-U6-*Apoe*(mouse)-shRNA-CopGFP-Puro and the non-targeting scramble control vector were purchased from Miaoling Biology (Wuhan, China). Both vectors carried CopGFP and puromycin resistance markers. After transduction, cells were selected with 10 μg/mL puromycin for 3–5 days to obtain stable *Apoe*-knockdown and matched control cells.

### Quantitative PCR and western blotting

*Apoe* knockdown in Hepa1–6 cells was validated by quantitative PCR and western blotting. Total RNA was extracted using the Eastep Super Total RNA Extraction Kit (Promega), and cDNA was synthesized using the Eastep RT Master Mix Kit. Quantitative PCR was performed with Eastep qPCR Master Mix. β-actin was used as the internal control, and relative *Apoe* expression was calculated using the 2^−ΔΔCt^ method (33). Primer sequences are listed in **Supplementary Table 6**.

For western blotting, ApoE was detected with an anti-ApoE rabbit monoclonal antibody (E7X2A, Cell Signaling Technology, #49285, 1:1000), and β-actin was detected with a β-actin rabbit monoclonal antibody (D6A8, Cell Signaling Technology, #8457S, 1:1000) as the loading control. Membranes were incubated with HRP-linked anti-rabbit IgG secondary antibody (Cell Signaling Technology, #7074S, 1:1000), and signals were detected using Super ECL Detection Reagent (36208ES60).

### Mouse subcutaneous tumor models

Seven-week-old male wild-type (WT) C57BL/6J, *Apoe*^−/−^ C57BL/6J, and NOD.Cg-*Prkdc*^scid^ *Il2rg*^tm1Sug^/JicCrl (NOG) mice were used. In the host *Apoe*-deficiency model, parental Hepa1–6 cells were injected subcutaneously into wild-type C57BL/6J and *Apoe*^−/−^ C57BL/6J mice (*n = 7* per group). In the tumor-cell *Apoe* knockdown model, control shRNA (sh-NC) or *Apoe*-targeting shRNA (sh-*Apoe*) Hepa1–6 cells were injected subcutaneously into NOG mice (*n = 6* per group). Each mouse received 1 × 10^6^ cells in the left axillary region. Tumors were collected on day 21 after implantation. Tumor volume was calculated from length and width measurements as length × width²/2, and tumor weight was recorded.

### *Nras-Myc*-driven liver tumor model

Male wild-type C57BL/6J mice and *Apoe*^−/−^ mice on the same genetic background were maintained under specific pathogen-free conditions with free access to standard chow and water. At 7 weeks of age, liver tumors were induced by hydrodynamic tail vein injection of a plasmid mixture containing PT/Caggs-NRas-V12, PT3-EF1a-C-Myc, and pCMV-SB11 according to the supplier’s protocol. The injection volume was 1 mL per 10 g body weight and was delivered evenly within 5–8 s. Plasmids and corresponding control vectors were purchased from Shouzheng Hongyao Biotechnology Co., Ltd. (Wuhan, China). Liver tissues were collected on day 42 after injection. Eight mice were included in each genotype group; at endpoint, three mice per group were used for single-cell analysis, and the remaining five mice per group were used for histological and immunostaining analyses.

### Mouse single-cell RNA sequencing analysis

Single-cell RNA sequencing was performed on liver tumor tissues from three WT and three *Apoe*^−/−^ mice maintained on a normal diet in the *Nras-Myc*-driven liver tumor model. Single-cell suspensions were processed using the 10x Genomics Chromium platform, and gene-expression libraries were prepared using the Single Cell 3′ Reagent Kit v3.1. Libraries were sequenced on an Illumina NovaSeq 6000 platform. Raw sequencing data were processed using Cell Ranger count v7.0.1 against the mouse mm10 reference genome (mm10-1.2.0) to generate filtered gene-barcode matrices. Mouse single-cell data were then processed in Seurat using quality control, normalization, highly variable gene selection, dimensionality reduction, clustering, and UMAP visualization. Batch effects among samples were corrected using Harmony. Major cell populations were annotated using canonical lineage markers, and myeloid cells were further extracted for reclustering and subtype annotation.

After myeloid reclustering, subtype annotation was refined using representative marker genes. Macrophage/monocyte functional programs were assessed using literature-informed curated gene modules related to lipid/cholesterol handling, lysosome/phagocytosis, inflammatory chemokines, and angiogenic/injury ligands. Module scores were summarized at the mouse-sample level for genotype-associated comparisons.

### Histological staining and image quantification

Tumor tissues were fixed in 4% paraformaldehyde, paraffin-embedded, and sectioned at 5 μm. Hematoxylin and eosin (HE) staining was performed for histological assessment of liver tumor tissues. CD8 immunohistochemistry was performed to assess CD8-positive cell infiltration using an anti-CD8 primary antibody (AiFang Biological, #AFRM0004, 1:150), followed by 3,3′-diaminobenzidine (DAB) visualization and hematoxylin counterstaining. Ki67 immunohistochemistry was performed to assess tumor-cell proliferation using an anti-Ki67 primary antibody (AiFang Biological, #AFRP0030, 1:600). CD31 immunofluorescence was performed to assess tumor vascularization using an anti-CD31 primary antibody (AiFang Biological, #AFRM0001, 1:500), with nuclei counterstained with 4′,6-diamidino-2-phenylindole (DAPI).

For CD8 and CD31 quantification in the Hepa1–6 subcutaneous tumor model, one tumor field was analyzed from each of seven mice per group. For Ki67 quantification in the *Nras-Myc*-driven liver tumor model, two non-necrotic fields were quantified from each of five tumors per group, and the mean value of the two fields was used as one mouse-level data point for statistical analysis. Images were quantified using ImageJ (34). CD8-positive cells were calculated as the percentage of CD8-positive cells among total cells, Ki67-positive nuclei were calculated as the percentage of Ki67-positive nuclei among total nuclei, and CD31-positive vascular area was calculated as the percentage of CD31-positive area within the analyzed field. Multiplex immunofluorescence analysis of liver tumor tissues was performed using a TSA-based staining protocol with primary antibodies against ApoE (#AB183597, 1:1000), CD86 (YM8023, 1:1000), and CD206 (#24595, 1:3000), followed by DAPI nuclear counterstaining.

### Bone marrow-derived macrophage culture and BODIPY 493/503 staining

Bone marrow cells from WT and *Apoe*^−/−^ mice were differentiated into bone marrow-derived macrophages (BMDMs) with macrophage colony-stimulating factor (M-CSF; 25 ng/mL; MedChemExpress, HY-P7085) for 7 days. BMDMs were left untreated or treated with oleic acid (OA; 200 μM; Xi’an Kunchuang Technology Development Co., Ltd., KC006) or human oxLDL (50 μg/mL; Yeasen Biotechnology, 20605ES) for 24 h. Cells were stained with BODIPY 493/503 (2 μM; MedChemExpress, HY-D1614) for 15 min at 37 °C, washed with PBS, and analyzed by flow cytometry. BODIPY 493/503 fluorescence was quantified as background-subtracted median fluorescence intensity (ΔMedian MFI) in gated BMDMs.

### Statistical analysis

Statistical analyses were performed using R version 4.5.0 and GraphPad Prism version 9.5. Single-cell analyses were performed using Seurat version 5.3.0, Harmony version 1.2.3, scMetabolism version 0.2.1, AUCell version 1.30.1, and CellChat version 2.1.2. Image quantification was performed using ImageJ. GraphPad Prism version 9.5 was used for statistical analyses of mouse tumor measurements, qPCR validation, immunostaining quantification, and BODIPY 493/503 flow-cytometry data from BMDMs. Paired comparisons, including tumor-versus-adjacent sample-level analyses, were performed using the paired Wilcoxon signed-rank test. For human samples, tumor and adjacent tissues from the same patients were compared as paired samples, without additional adjustment for patient covariates. Comparisons between two independent groups were performed using two-tailed unpaired Student’s t-tests for data with comparable variances, Welch’s t-tests when variances were unequal, or Wilcoxon rank-sum tests, also referred to as Mann–Whitney U tests, for nonparametric comparisons, as appropriate. BODIPY 493/503 signals in BMDMs were compared between WT and *Apoe*^−/−^ groups using unpaired two-tailed Welch’s t-tests. Mouse single-cell composition and module-score analyses were summarized at the mouse-sample level.

Multiple comparisons were adjusted using the Benjamini–Hochberg method where applicable (35). All tests were two-sided, and *P < 0.05* was considered statistically significant.

## Results

### Single-cell profiling reveals immune-metabolic remodeling in HCC

**Figure 1:**
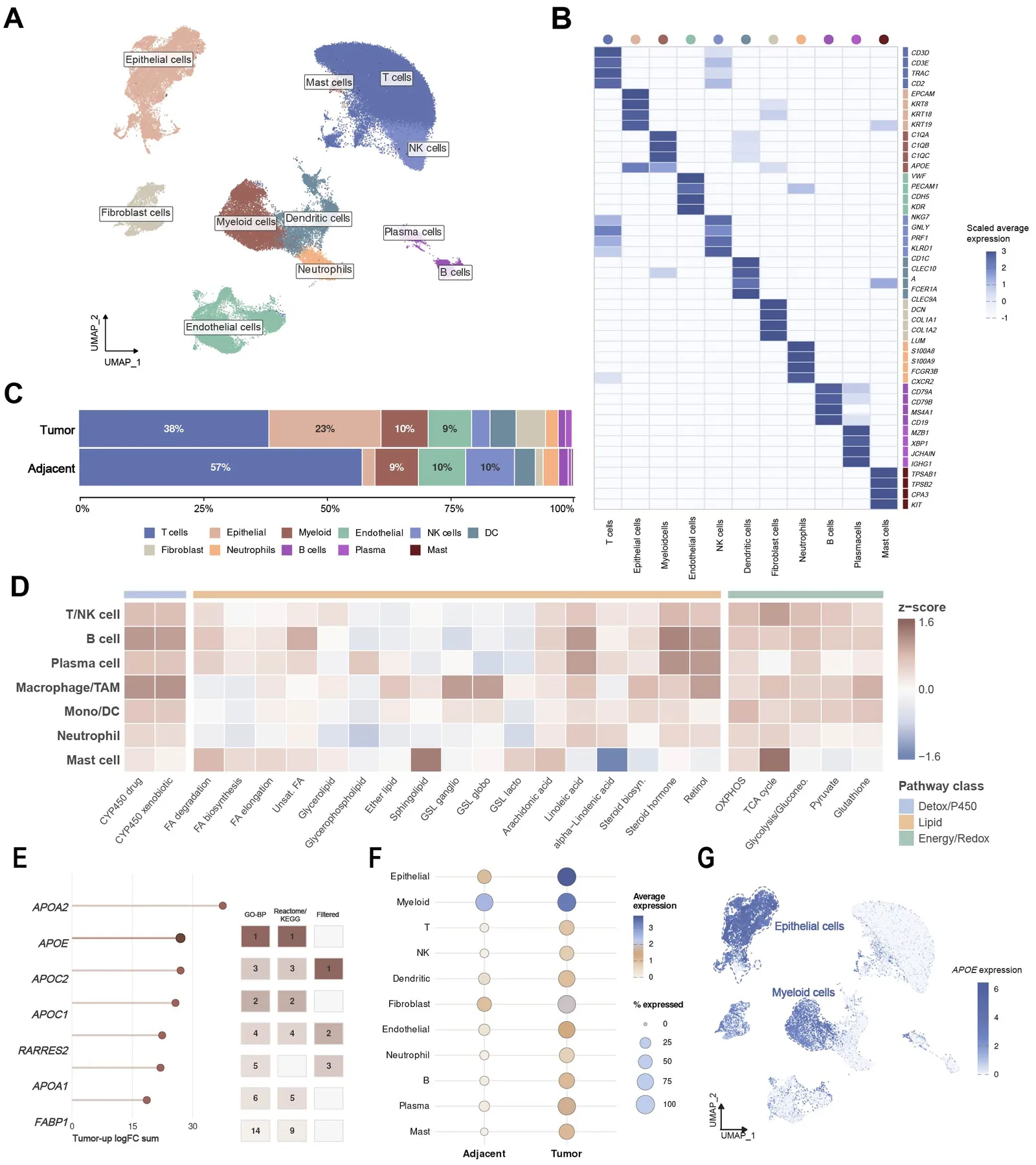
Single-cell profiling and lipid-related candidate screening in HCC. **(A)** UMAP isualization of the integrated single-cell dataset from paired HCC tumor and tumor-adjacent liver tissues, showing the major annotated cell populations. **(B)** Heatmap of canonical lineage marker expression across annotated cell types. **(C)** Stacked bar plot showing major cell fractions in tumor and adjacent tissues. **(D)** Heatmap showing tumor-associated changes in metabolic pathway activity across immune cell groups. Values indicate tumor-minus-adjacent differences in sample-level z-scored pathway activity. **(E)** Lipid-related tumor-upregulated candidate genes ranked by summed tumor-upregulated log fold change across immune-cell populations, with tile annotations showing gene ranks under lipid-related GO biological process terms, Reactome/KEGG pathway filters, and the focused retained-gene filter. Blank tiles indicate genes not ranked or not retained under the corresponding filtering strategy. **(F)** Dot plot showing *APOE* expression across major cell types in adjacent and tumor tissues. Dot size indicates the percentage of cells expressing *APOE*, and color indicates scaled average expression. **(G)** Feature plot showing *APOE* expression across the integrated single-cell dataset. T/NK, T and natural killer cells; Mono/DC, monocyte/dendritic-cell compartment.

We analyzed single-cell transcriptomic profiles from 10 paired HCC tumors and adjacent liver tissues to characterize tumor-associated changes in immune composition and metabolic activity. After quality control, batch correction, clustering, and marker-based annotation, the integrated atlas identified major cell populations, including epithelial cells, T cells, myeloid cells, endothelial cells, NK cells, dendritic cells, fibroblasts, neutrophils, B cells, plasma cells, and mast cells (**Figure 1A**). These annotations were supported by canonical lineage markers shown in **Figure 1B**.

Tumor tissues differed from adjacent liver tissues in both cell composition and metabolic pathway activity. At the compositional level, tumors showed marked epithelial enrichment and a lower T-cell fraction, while myeloid cells remained a substantial component of the tumor microenvironment (**Figure 1C**). We then calculated tumor-minus-adjacent z-score differences in metabolic pathway activity across major immune cell groups. The heatmap showed tumor-associated shifts in CYP450/xenobiotic detoxification, lipid metabolism, and energy/redox pathways, with lineage-specific differences in direction and magnitude (**Figure 1D**). Together, these findings show that HCC tumors are characterized by coordinated changes in immune-cell composition and metabolic pathway activity, providing the basis for screening tumor-associated lipid-handling factors.

### *APOE* is prioritized as a tumor-associated lipid-handling factor

We then asked which tumor-upregulated lipid-related genes could link the immune-metabolic remodeling observed above to specific cellular programs in HCC. Across immune-cell populations, candidate genes were ranked by summed tumor-upregulated log fold change and further evaluated using lipid-related GO biological process terms, Reactome/KEGG pathways, and a focused retained-gene filter. This strategy highlighted a set of apolipoprotein and lipid-handling genes, including *APOA2, APOE, APOC2, APOC1, RARRES2, APOA1*, and *FABP1* (**Figure 1E**). *APOE* was consistently retained across lipid-related screening strategies and ranked first under the focused lipid-related filter, supporting its selection for further evaluation as a tumor-associated lipid-handling candidate.

*APOE* expression was not restricted to a single lineage; in tumor samples, it was relatively prominent in the epithelial compartment and in myeloid cells (**Figures 1F, G**). This dual cellular distribution suggested that *APOE* may have cell-type-dependent roles in the HCC tumor microenvironment. Given that ApoE is a secreted apolipoprotein, this expression pattern also provided a rationale for examining candidate ligand–receptor communication involving *APOE-*positive macrophages. We therefore focused subsequent analyses on the myeloid compartment to determine whether *APOE* marked a tumor-enriched macrophage state in HCC.

### Myeloid reclustering identifies tumor-enriched *APOE-*positive macrophages with lipid-associated programs

**Figure 2:**
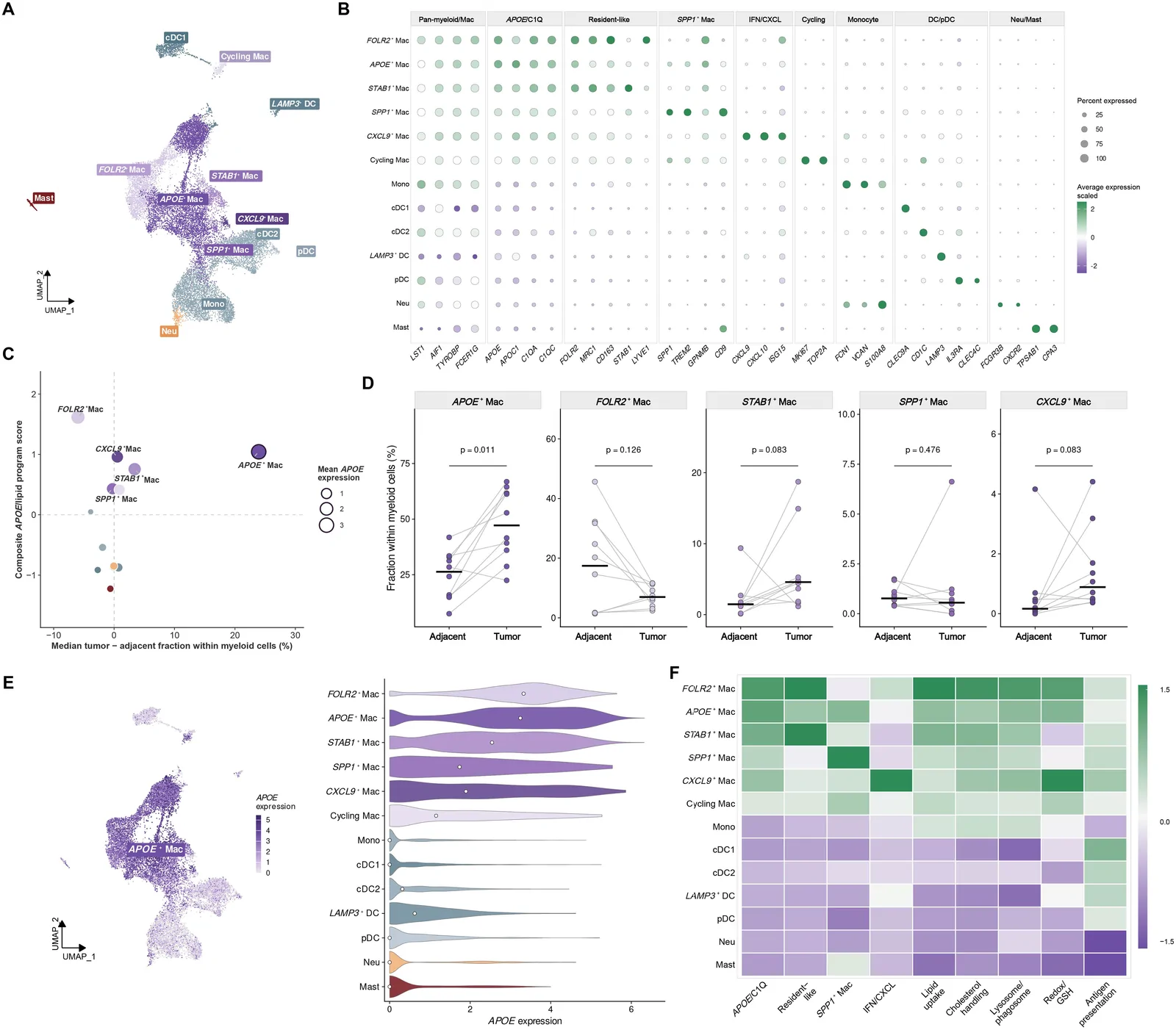
Myeloid reclustering identifies tumor-enriched *APOE-*positive macrophages. **(A)** UMAP visualization of reclustered myeloid cells, showing macrophage, monocyte, dendritic-cell, neutrophil, and mast-cell populations. **(B)** Dot plot showing marker genes used for myeloid-state annotation. Dot size indicates the percentage of cells expressing each gene, and color indicates scaled average expression. **(C)** Bubble plot integrating tumor-associated enrichment, *APOE/*lipid program activity, and *APOE* expression across myeloid states. The x-axis shows the median tumor-minus-adjacent fraction within myeloid cells, the y-axis shows the composite *APOE/*lipid program score, and bubble size indicates mean *APOE* expression. **(D)** Paired sample-level comparison of selected macrophage-state fractions within myeloid cells between adjacent and tumor tissues. Each line connects one paired patient sample (*n = 10* pairs); horizontal bars indicate group medians. *P* values were calculated using paired Wilcoxon signed-rank tests. **(E)** Feature plot and violin plot showing *APOE* expression across reclustered myeloid states. **(F)** Heatmap showing functional module scores across myeloid states, including *APOE*/C1Q, resident-like, *SPP1*-positive macrophage, IFN/CXCL, lipid uptake, cholesterol handling, lysosome/phagosome, redox/glutathione (GSH), and antigen-presentation programs.

To define the myeloid component of the *APOE-*associated signal observed in the global atlas, we extracted myeloid cells and performed reclustering. This analysis separated several macrophage states, including *FOLR2*-positive, *APOE*-positive, *STAB1*-positive, *SPP1*-positive, *CXCL9*-positive, and cycling macrophages, together with monocytes, dendritic-cell subsets, neutrophils, and mast cells (**Figure 2A**). Canonical lineage and state-associated markers supported these annotations and distinguished *APOE-*positive macrophages from resident-like, *SPP1*-positive, interferon/CXCL, dendritic-cell, monocyte, neutrophil, and mast-cell populations (**Figure 2B**).

We then used three complementary criteria to prioritize myeloid states for downstream analysis: tumor-associated enrichment, mean *APOE* expression, and composite *APOE/*lipid-program activity. *APOE-*positive macrophages showed the clearest convergence of these criteria, with a positive tumor-minus-adjacent fraction shift, high *APOE* expression, and elevated *APOE/*lipid-program activity compared with other myeloid states (**Figure 2C**). Paired sample-level analysis further showed that *APOE-*positive macrophages were significantly increased in tumors (*P = 0.011*), whereas the other selected macrophage states showed weaker or nonsignificant tumor-associated changes (**Figure 2D**).

**Figure 3:**
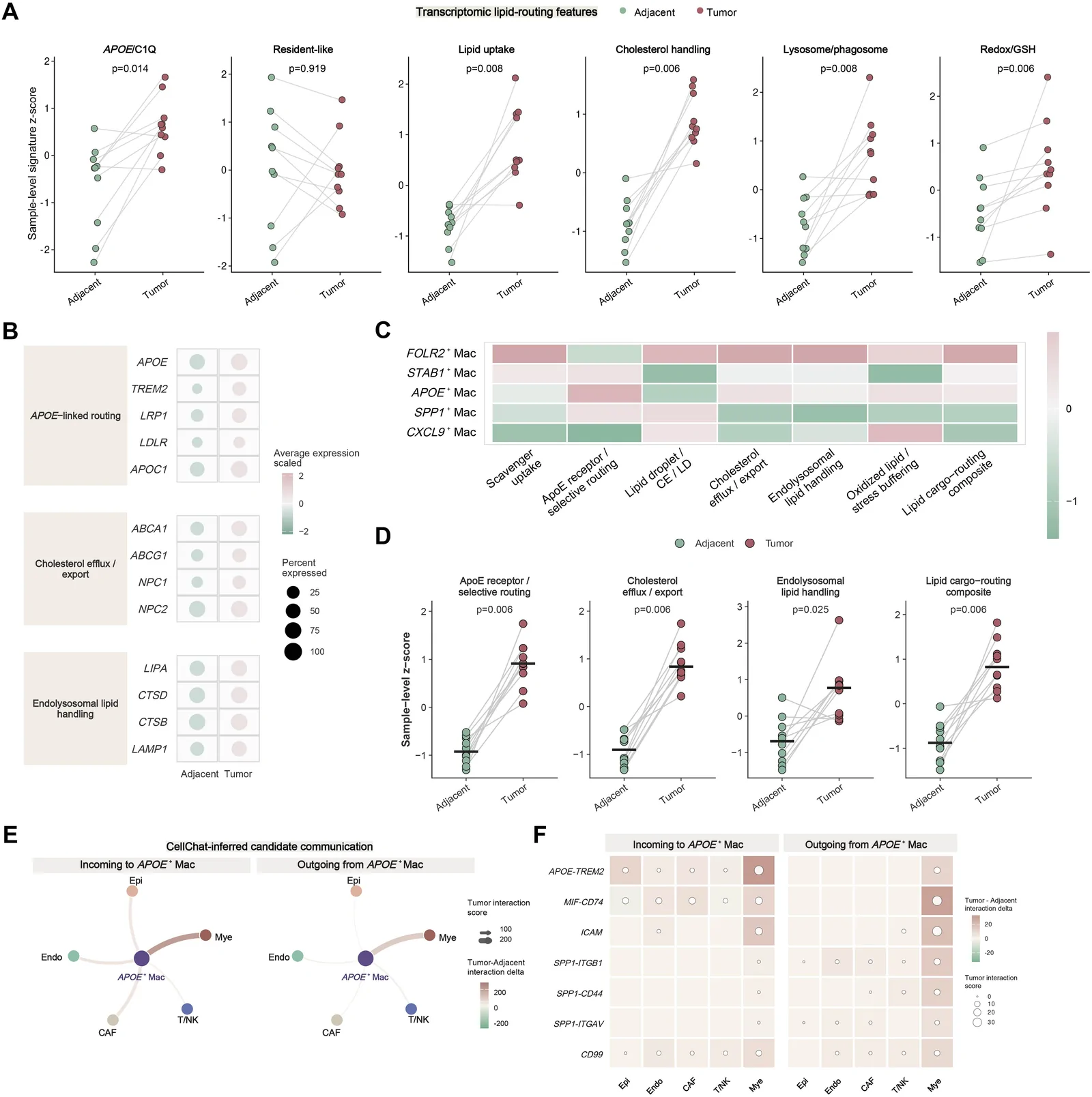
Tumor *APOE-*positive macrophages display transcriptomic lipid-routing features and CellChat-inferred candidate communication axes. **(A)** Paired patient-sample comparison of module z-scores in adjacent and tumor *APOE*-positive macrophages, including *APOE*/C1Q, resident-like, lipid uptake, cholesterol handling, lysosome/phagosome, and redox/GSH programs. Each line connects paired samples. *P* values were calculated using paired Wilcoxon signed-rank tests. **(B)** Dot plot showing genes involved in *APOE-*linked routing, cholesterol efflux/export, and endolysosomal lipid handling in adjacent and tumor *APOE-*positive macrophages. Dot size indicates the percentage of cells expressing each gene, and color indicates scaled average expression. **(C)** Heatmap showing lipid cargo-routing module scores across macrophage states, including scavenger uptake, ApoE receptor/selective routing, lipid droplet (LD)/cholesteryl ester (CE)-related activity, cholesterol efflux/export, endolysosomal lipid handling, oxidized lipid/stress buffering, and the lipid cargo-routing composite. **(D)** Paired patient-sample comparison of ApoE receptor/selective routing, cholesterol efflux/export, endolysosomal lipid handling, and lipid cargo-routing composite z-scores in adjacent and tumor *APOE*-positive macrophages. Each line connects paired samples; horizontal bars indicate group medians. *P* values were calculated using paired Wilcoxon signed-rank tests. **(E)** CellChat-inferred candidate incoming and outgoing interaction networks involving *APOE-*positive macrophages. Edge width indicates tumor interaction score, and edge color indicates tumor-minus-adjacent interaction delta. **(F)** Dot plot of selected CellChat-inferred incoming and outgoing ligand–receptor interactions involving *APOE*-positive macrophages. Dot size indicates tumor interaction score, and color indicates tumor-minus-adjacent interaction delta. The selected ligand–receptor pairs shown in **(F)** are listed in **Supplementary Table 5**. For paired patient-sample comparisons in **(A, D)**, *n = 10* paired patient samples. Epi, epithelial cells; Endo, endothelial cells; CAF, fibroblast/cancer-associated fibroblast; T/NK, T and natural killer cells; Mye, other myeloid cells.

*APOE* feature visualization and violin plots confirmed that *APOE* expression within the reclustered myeloid compartment was concentrated mainly in macrophage states, with the strongest signal in *APOE-*positive macrophages (**Figure 2E**). Functional module scoring further showed that this population carried high *APOE*/C1Q activity and relatively high lipid uptake, cholesterol handling, lysosome/phagosome, and redox/glutathione (GSH)-related scores, whereas antigen-presentation activity was more prominent in dendritic-cell subsets (**Figure 2F**). Together, these analyses identified *APOE-*positive macrophages as the myeloid population that best matched our selection criteria, including tumor enrichment, high *APOE* expression, and lipid-associated macrophage programs. We therefore focused on this population for the analyses in **Figure 3**.

### Tumor *APOE-*positive macrophages show coordinated lipid routing, cholesterol trafficking, and endolysosomal processing

We next characterized the lipid-processing program of tumor-enriched *APOE*-positive macrophages. In paired patient-sample analysis, tumor *APOE-*positive macrophages showed higher *APOE/*C1Q module activity than adjacent *APOE-*positive macrophages (*P = 0.014*), whereas the resident-like module did not differ significantly (*P = 0.919*). Tumor *APOE*-positive macrophages also showed increased lipid uptake (*P = 0.008*), cholesterol handling (*P = 0.006*), lysosome/phagosome activity (*P = 0.008*), and redox/GSH-related activity (*P = 0.006*) (**Figure 3A**). These paired comparisons indicated that tumor *APOE-*positive macrophages were characterized by lipid-handling, lysosome/phagosome, and redox-adaptive activity, without a dominant increase in the resident-like program.

At the gene level, tumor *APOE-*positive macrophages showed higher expression of representative genes involved in apolipoprotein-associated lipid handling (*APOE* and *APOC1*), receptor-linked lipid routing (*TREM2, LRP1*, and *LDLR*), cholesterol trafficking or efflux (*ABCA1, ABCG1, NPC1*, and *NPC2*), and endolysosomal processing (*LIPA, CTSD, CTSB*, and *LAMP1*) (**Figure 3B**). This pattern was consistent with a coordinated program of lipoprotein recognition, receptor-associated lipid routing, cholesterol trafficking, and lysosomal processing.

We then compared lipid cargo-routing programs across macrophage states. *APOE-*positive macrophages showed a pattern distinct from *FOLR2*-positive, *STAB1*-positive, *SPP1*-positive, and *CXCL9*-positive macrophages, with relative enrichment of ApoE receptor/selective routing and a positive lipid cargo-routing composite score (**Figure 3C**). Paired sample-level comparisons further confirmed higher ApoE receptor/selective routing activity *(P = 0.006*), cholesterol efflux/export activity (*P = 0.006*), endolysosomal lipid-handling activity (*P = 0.025*), and lipid cargo-routing composite score (*P = 0.006*) in tumor *APOE-*positive macrophages than in adjacent *APOE-*positive macrophages (**Figure 3D**). Together, these findings define *APOE*-positive macrophages as a tumor-enriched lipid-routing state characterized by coordinated cholesterol trafficking, endolysosomal processing, and redox adaptation.

### CellChat analysis identifies candidate ligand–receptor interactions involving *APOE-*positive macrophages

To nominate candidate cell–cell communication routes involving *APOE*-positive macrophages, we performed CellChat analysis comparing tumor and adjacent tissues. We focused on inferred incoming and outgoing ligand–receptor interactions involving *APOE-*positive macrophages, and summarized these interactions using tumor interaction scores and tumor-minus-adjacent deltas. This analysis first provided a network-level view of *APOE-*positive macrophages as both receivers and senders in the tumor microenvironment (**Figure 3E**).

At the ligand–receptor level, the most prominent incoming candidate interaction toward *APOE-*positive macrophages was *APOE–TREM2*, primarily from the epithelial compartment. Additional inferred *APOE–TREM2* signals were detected from fibroblast/cancer-associated fibroblast (CAF), other myeloid, endothelial, and T and natural killer (T/NK) compartments. Other incoming signals toward *APOE-*positive macrophages included *MIF–CD74, ICAM*-related, and *CD99*-related interactions. In the outgoing direction, *APOE-*positive macrophages showed inferred interactions involving *MIF–CD74, ICAM*-related, *CD99-*related, and *SPP1-*associated pairs, including *SPP1–ITGB1, SPP1–CD44*, and *SPP1–ITGAV* (**Figure 3F**). These results nominate candidate communication routes involving *APOE-*positive macrophages, including lipid receptor-related, inflammatory/immune, adhesion/migration, and *SPP1-*associated signals in the HCC tumor microenvironment.

### Host *Apoe* deficiency attenuates tumor growth, whereas tumor-cell *Apoe* knockdown does not significantly affect tumor growth in NOG mice

**Figure 4:**
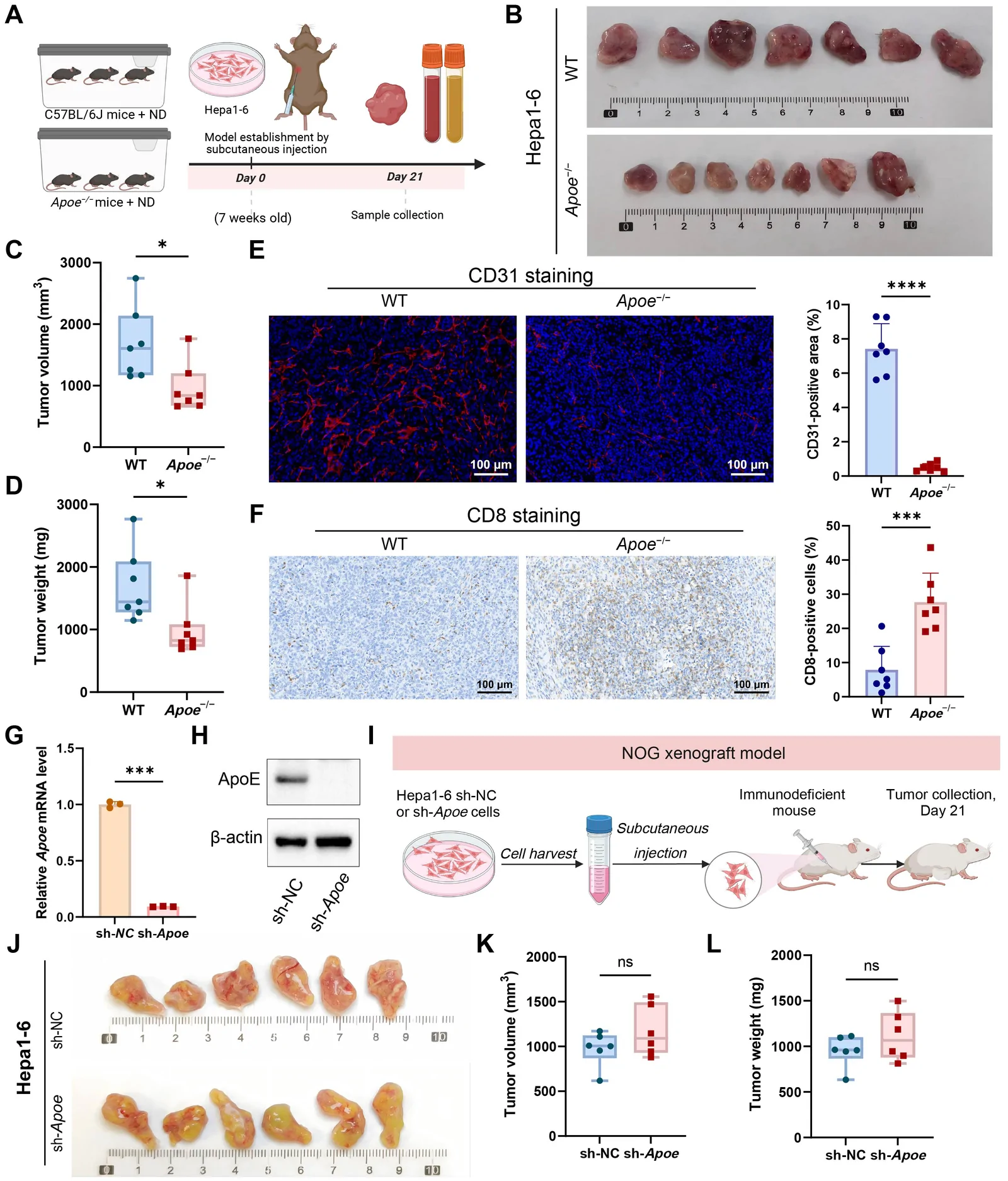
Host *Apoe* deficiency attenuates Hepa1–6 tumor growth, whereas tumor-cell *Apoe* knockdown does not significantly affect tumor growth in NOG mice. **(A)** Experimental design for Hepa1–6 subcutaneous implantation in seven-week-old immunocompetent WT and *Apoe*^−/−^ C57BL/6J mice. **(B)** Gross images of endpoint tumors from WT and *Apoe*^−/−^ hosts. **(C,D)** Quantification of tumor volume **(C)** and tumor weight **(D)** in WT and *Apoe*^−/−^ hosts. **(E)** Representative CD31 immunofluorescence images and quantification of CD31-positive vascular area. **(F)** Representative CD8 immunohistochemistry images and quantification of CD8-positive cells. **(G, H)** Validation of *Apoe* knockdown in Hepa1–6 cells by quantitative PCR **(G)** and western blotting **(H, I)** Experimental design for the NOG xenograft model using sh-NC or sh-*Apoe* Hepa1–6 cells in seven-week-old NOG mice. Tumors were collected on day 21 after subcutaneous implantation. **(J)** Gross images of endpoint tumors from the NOG xenograft model. **(K, L)** Quantification of tumor volume **(K)** and tumor weight **(L)** in sh-NC and sh-*Apoe* groups. For the host *Apoe*-deficiency model, *n = 7* mice per group, and one tumor field was quantified from each mouse for CD31 and CD8 analyses. For the NOG xenograft model, *n = 6* mice per group. For qPCR validation in G, *n = 3* independent cell samples per group. *P* values in **(C, D)** were calculated using two-tailed Mann–Whitney U tests; *P* values in **(E, G)** were calculated using two-tailed Welch’s t-tests; and *P* values in **(F, K, L)** were calculated using two-tailed unpaired Student’s t-tests. Scale bars in **(E, F)**, 100 μm. ns, not significant; *P < 0.05; ***P < 0.001; ****P < 0.0001.

To determine whether host *Apoe* deficiency affected tumor growth *in vivo*, we first established a subcutaneous Hepa1–6 model in immunocompetent WT and *Apoe-*deficient mice maintained on a normal diet (**Figure 4A**). Compared with WT hosts, *Apoe-*deficient hosts developed smaller endpoint tumors (**Figure 4B**), with reduced tumor volume and tumor weight (**Figures 4C, D**). These findings suggest that host *Apoe* contributes to Hepa1–6 tumor growth in an immunocompetent setting.

We next examined vascularization and CD8*-*positive cell infiltration in these tumors. Tumors from *Apoe-*deficient mice showed markedly reduced CD31*-*positive vascular area and increased CD8*-*positive cell infiltration compared with tumors from WT mice (**Figures 4E, F**), indicating that host *Apoe* deficiency was associated with reduced tumor vascularization and increased CD8*-*positive immune-cell infiltration.

We next tested whether tumor-cell *Apoe* knockdown altered tumor growth under immunodeficient conditions using stable *Apoe*-knockdown Hepa1–6 cells. Quantitative PCR and western blotting confirmed reduced *Apoe* mRNA and ApoE protein expression compared with sh-NC controls (**Figures 4G, H**). After implantation into immunodeficient NOG mice (**Figure 4I**), sh-*Apoe* tumors showed no significant changes in tumor volume or tumor weight compared with sh-NC tumors (**Figures 4J–L**). Together, these findings support a host *Apoe*-dependent contribution to Hepa1–6 tumor growth in an immunocompetent setting, while tumor-cell *Apoe* knockdown produced no significant change in tumor volume or tumor weight in the immunodeficient NOG model.

### Host *Apoe* deficiency attenuates *Nras-Myc*-driven liver tumor development

**Figure 5:**
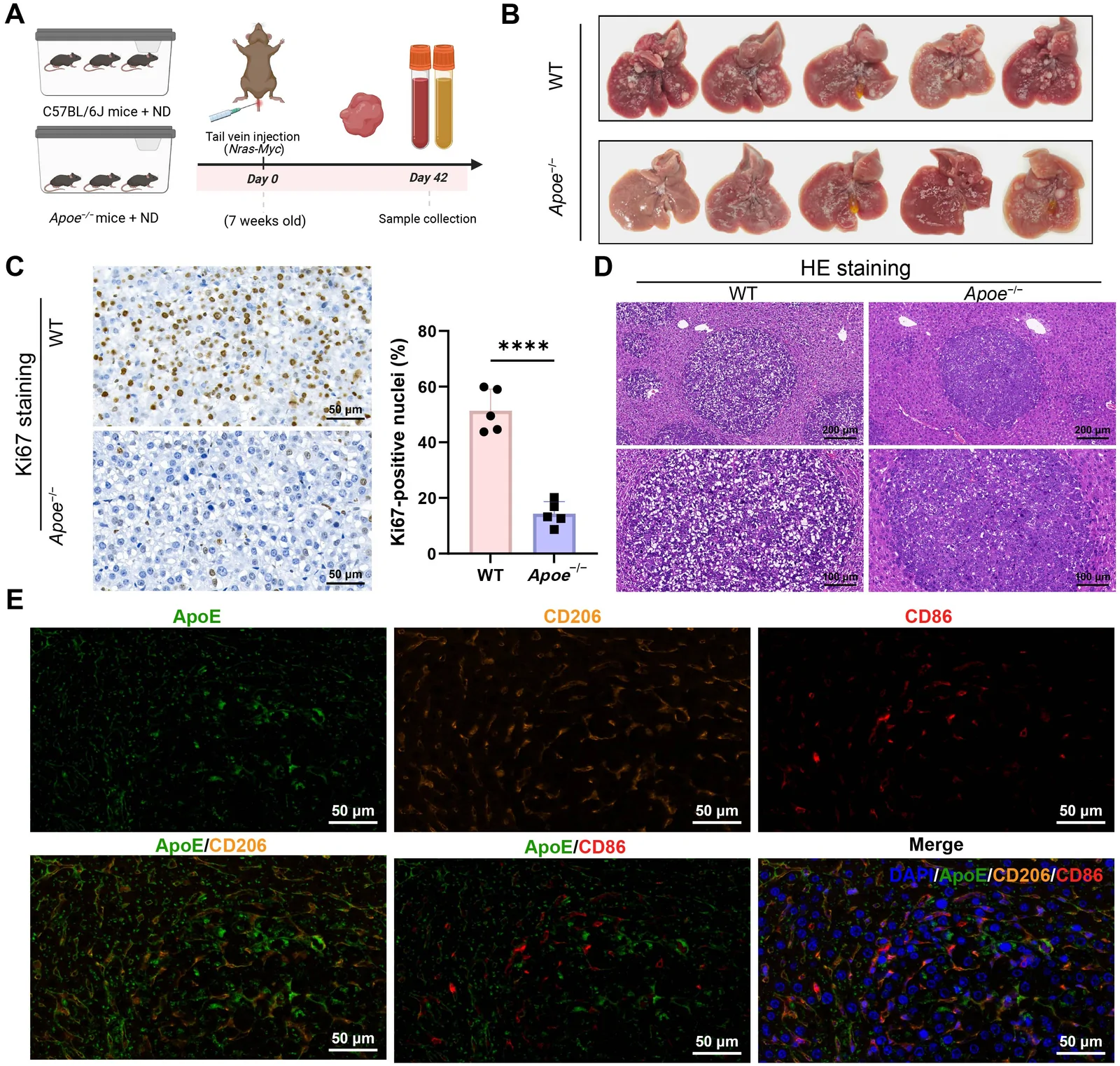
Host *Apoe* deficiency attenuates *Nras-Myc*-driven liver tumor development. **(A)** Experimental design of the *Nras-Myc*-driven liver tumor model in seven-week-old WT and *Apoe*^−/−^ C57BL/6J mice. Liver tumors were induced by hydrodynamic tail vein injection, and liver tissues were collected on day 42. **(B)** Gross liver images from WT and *Apoe*^−/−^ mice at sample collection. **(C)** Representative Ki67 immunohistochemistry images and quantification of Ki67-positive nuclei in WT and *Apoe*^−/−^ liver tumors. **(D)** Representative HE staining images of liver tumor tissues from WT and *Apoe*^−/−^ mice. **(E)** Representative multiplex immunofluorescence images showing ApoE, CD206, CD86, and merged staining in WT liver tumor tissue. For histological and immunostaining analyses, *n = 5* mice per group. For Ki67 quantification, two non-necrotic fields were quantified from each of five tumors per group, and the mean value of the two fields was used as one mouse-level data point. The *P* value in **(C)** was calculated using a two-tailed unpaired Student’s t-test. Scale bars are indicated in **(C–E)**. ****P < 0.0001.

We next examined whether a similar reduction in tumor development was observed in a liver tumor model with host *Apoe* deficiency. For this purpose, we used an *Nras-Myc*-driven liver tumor model in WT and *Apoe-*deficient mice maintained on a normal diet (**Figure 5A**). Compared with WT mice, *Apoe-*deficient mice showed a lower gross liver tumor burden at sample collection (**Figure 5B**). Histological assessment further showed reduced tumor-cell proliferation in *Apoe-*deficient livers, as indicated by a lower percentage of Ki67-positive nuclei (**Figure 5C**). HE staining also showed fewer tumor-associated histological lesions in *Apoe-*deficient mice than in WT mice (**Figure 5D**). Multiplex immunofluorescence was used to examine the protein-level distribution of ApoE in WT liver tumor tissue. In representative images, ApoE signal was detectable in the tumor microenvironment and was more apparent in CD206-positive macrophage-rich areas than in CD86-positive areas (**Figure 5E**).

### *Apoe* deficiency is associated with changes in tumor myeloid states and functional programs in *Nras-Myc* liver tumors

**Figure 6:**
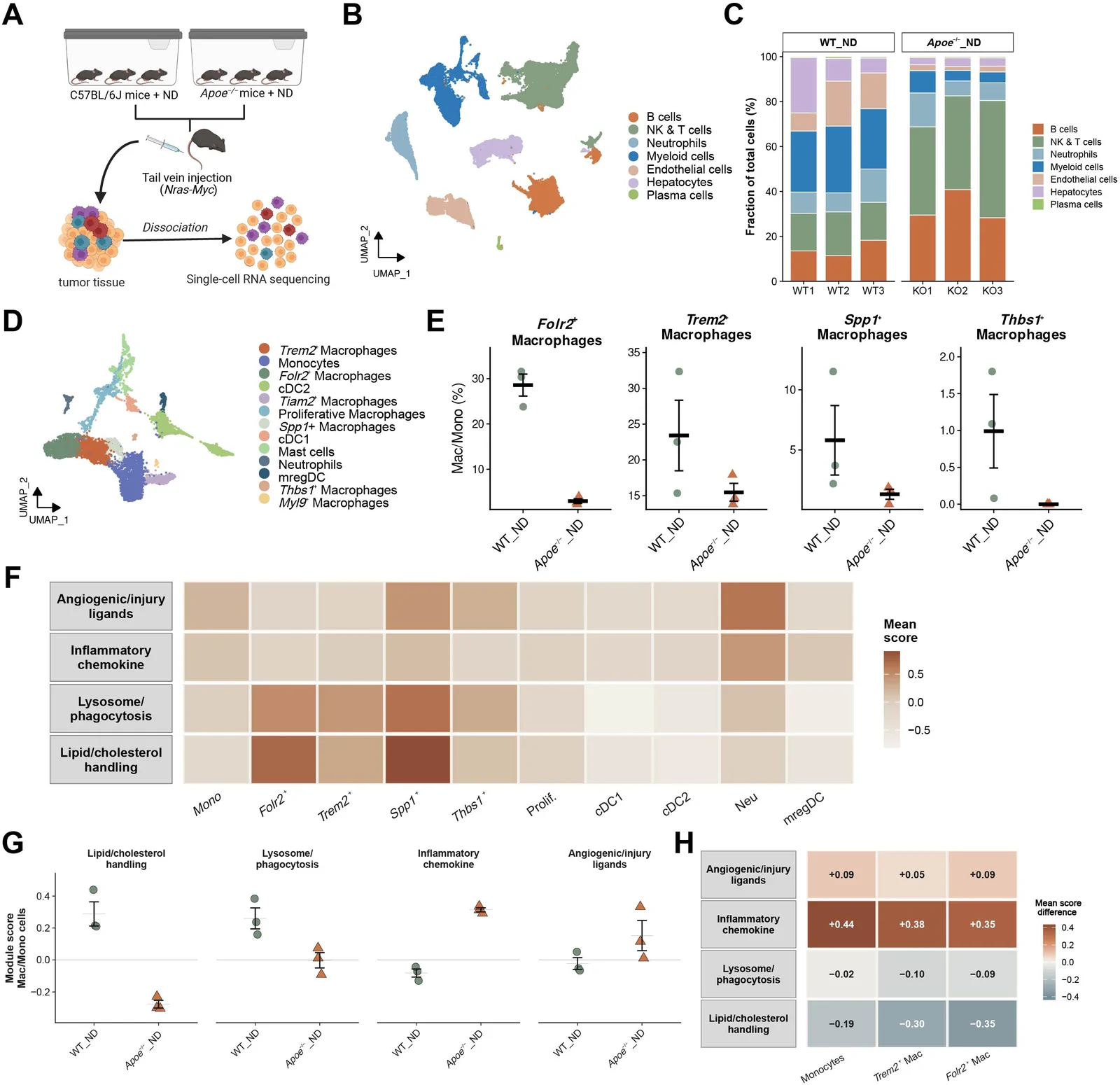
*Apoe* deficiency is associated with changes in tumor myeloid states and functional programs in *Nras-Myc* liver tumors. **(A)** Experimental design for single-cell RNA sequencing of liver tumor tissues from WT and *Apoe*^−/−^ mice in which *Nras-Myc* tumors were induced at 7 weeks of age. Tumor tissues were collected on day 42 after hydrodynamic tail vein injection. **(B)** UMAP visualization of the integrated mouse tumor single-cell dataset, showing major annotated cell populations. **(C)** Stacked bar plot showing major cell-type fractions in individual WT and *Apoe*^−/−^ tumor samples. Each bar represents one mouse sample. WT1–WT3 and KO1–KO3 denote individual mice within each genotype group. **(D)** UMAP visualization of reclustered myeloid cells, showing macrophage, monocyte, dendritic-cell, neutrophil, and mast-cell populations. **(E)** Sample-level quantification of selected macrophage-state fractions within Mac/Mono cells in WT and *Apoe*^−/−^ tumors. Dots indicate individual mouse samples, and horizontal bars indicate mean ± standard error of the mean (SEM). **(F)** Heatmap showing functional module scores across myeloid states, including angiogenic/injury ligands, inflammatory chemokine, lysosome/phagocytosis, and lipid/cholesterol handling programs. **(G)** Comparison of functional module scores in Mac/Mono cells between WT and *Apoe*^−/−^ tumors. Dots indicate individual mouse samples, and horizontal bars indicate mean ± SEM. **(H)** State-level heatmap showing *Apoe*^−/−^ minus WT differences in functional module scores in monocytes, *Trem2*-positive macrophages, and *Folr2*-positive macrophages. For mouse single-cell RNA sequencing, *n = 3* mice per genotype. Mac/Mono, macrophage/monocyte compartment.

To characterize tumor microenvironmental changes associated with host *Apoe* deficiency, we performed single-cell transcriptomic analysis of *Nras-Myc* liver tumors from WT and *Apoe-*deficient mice maintained on a normal diet (**Figure 6A**). The integrated atlas identified major tumor microenvironmental populations, including B cells, NK and T cells, neutrophils, myeloid cells, endothelial cells, hepatocytes, and plasma cells (**Figure 6B**). Sample-level composition analysis showed genotype-associated differences between WT and *Apoe*-deficient tumors, suggesting that host *Apoe* loss was accompanied by changes in tumor microenvironmental composition (**Figure 6C**).

We then focused on the myeloid compartment. Myeloid reclustering identified monocytes, dendritic-cell subsets, neutrophils, and multiple macrophage states, including *Folr2*-positive, *Trem2*-positive, *Spp1-*positive, and *Thbs1*-positive macrophages (**Figure 6D**). Quantification at the sample level showed that *Apoe-*deficient tumors had reduced proportions of selected macrophage states, most notably *Folr2*-positive and *Trem2*-positive macrophages, with additional decreases in *Spp1*-positive and *Thbs1*-positive macrophages (**Figure 6E**). Functional module analysis showed that these macrophage states were linked to lipid/cholesterol handling and lysosome/phagocytosis programs (**Figure 6F**). When macrophages and monocytes were analyzed together, *Apoe-*deficient tumors showed lower lipid/cholesterol-handling and lysosome/phagocytosis scores, accompanied by higher inflammatory chemokine and angiogenic/injury ligand scores (**Figure 6G**). State-level delta analysis further showed reduced lipid/cholesterol handling and increased inflammatory chemokine scores within monocytes, *Trem2*-positive macrophages, and *Folr2*-positive macrophages (**Figure 6H**). Together, these mouse sample-level single-cell analyses suggest that host *Apoe* deficiency is associated with changes in tumor myeloid states and a shift in macrophage and monocyte functional programs in *Nras-Myc* liver tumors.

### *Apoe* deficiency increases neutral lipid accumulation in lipid-loaded BMDMs

**Figure 7:**
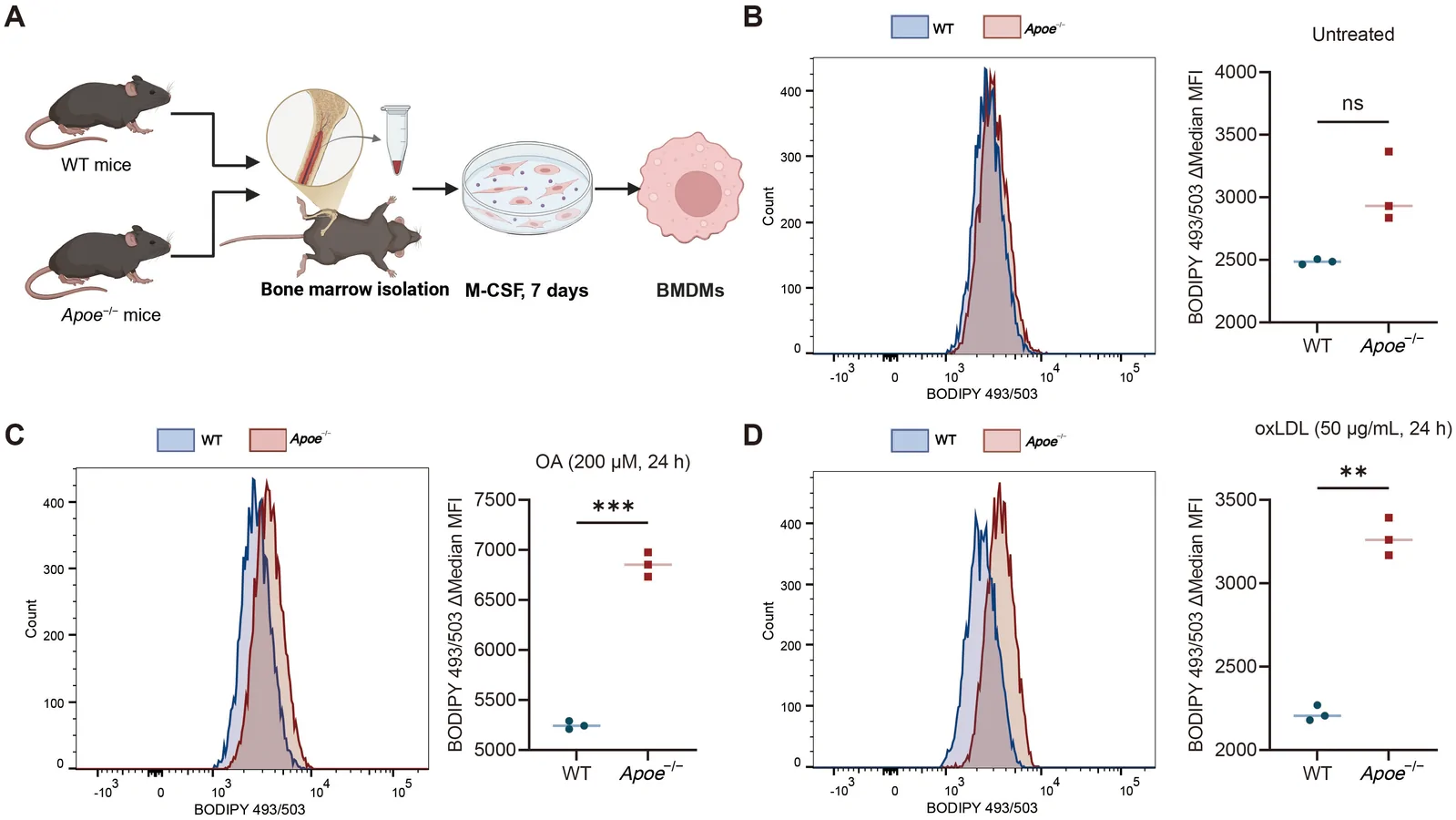
*Apoe* deficiency increases neutral lipid accumulation in lipid-loaded BMDMs **(A)** Schematic overview of BMDM isolation and BODIPY 493/503 staining. Bone marrow cells were isolated from WT and *Apoe*^−/−^ mice and differentiated into BMDMs with M-CSF for 7 days. **(B–D)** Representative flow cytometry histograms and quantification of BODIPY 493/503 signal in WT and *Apoe*^−/−^ BMDMs under untreated conditions **(B)**, after OA treatment (200 μM, 24 h) **(C)**, and after oxLDL treatment (50 μg/mL, 24 h) **(D)**. BODIPY 493/503 fluorescence was quantified as background-subtracted median fluorescence intensity (ΔMedian MFI). Data are shown as mean ± SEM. *n = 3* biological replicates per group. *P* values were calculated using unpaired two-tailed Welch’s t-tests. ns, not significant; **P < 0.01; ***P < 0.001. OA, oleic acid; oxLDL, oxidized low-density lipoprotein; M-CSF, macrophage colony-stimulating factor; BMDMs, bone marrow-derived macrophages.

To further test whether *Apoe* loss affects macrophage lipid handling at the cellular level, we performed BODIPY 493/503 staining in WT and *Apoe*^−/−^ BMDMs (**Figure 7A**). Under untreated conditions, BODIPY 493/503 signal did not differ significantly between WT and *Apoe*^−/−^ BMDMs (**Figure 7B**). After lipid loading with oleic acid (OA; 200 μM, 24 h) or oxLDL (50 μg/mL, 24 h), *Apoe*^−/−^ BMDMs showed higher BODIPY 493/503 signals than WT BMDMs (**Figures 7C, D**). These results indicate that *Apoe* deficiency enhances neutral lipid accumulation in BMDMs in response to lipid loading.

## Discussion

In HCC, chronic liver injury, lipid metabolism, immune regulation, and stromal remodeling together shape the tumor microenvironment (8). Macrophages are closely linked to this setting because they handle lipid and cholesterol cargo, clear dying cells, respond to inflammatory signals, and communicate with tumor, parenchymal, endothelial, and stromal cells (10, 11). How lipid-handling macrophage programs are connected to tumor-associated communication and host support in HCC remains unclear. Consistent with this concept, recent studies in other tumor contexts have shown that tumor-derived or tumor-associated signals can reshape macrophage functional states and influence antitumor immunity, further supporting the importance of macrophage-centered communication in the tumor microenvironment (36–38).

Here, we identified *APOE-*positive macrophages as a tumor-enriched myeloid state characterized by lipid routing, cholesterol handling, endolysosomal processing, and redox adaptation. CellChat analysis suggested candidate interactions between these macrophages and other tumor microenvironmental cells, including lipid receptor-related, immune/adhesion, and *SPP1*-associated signals. *In vivo*, host *Apoe* deficiency attenuated Hepa1–6 subcutaneous tumor growth and *Nras-Myc*-driven liver tumor development, whereas tumor-cell *Apoe* knockdown did not significantly affect tumor volume or tumor weight in NOG mice. In *Nras-Myc* tumors, this was accompanied by a contraction of lipid-processing macrophage states, particularly *Trem2*-positive and *Folr2*-positive macrophages, and by a shift in macrophage and monocyte programs from lipid handling and phagolysosomal activity toward inflammatory chemokine signaling. BODIPY 493/503 staining showed increased fluorescence in *Apoe*-deficient BMDMs after oleic acid or oxLDL loading, consistent with enhanced neutral lipid accumulation under lipid-loading conditions. These findings support a host microenvironmental role for *Apoe* in HCC growth and link *APOE*-associated macrophage lipid handling to myeloid remodeling in the tumor microenvironment.

*APOE* provides a genetic and functional entry point for understanding lipid-adapted macrophage programs in HCC. Germline studies have linked *APOE* locus variation to HCC susceptibility, placing *APOE* biology among host-level determinants that may influence liver carcinogenesis (22, 23). Functionally, ApoE participates in lipoprotein transport, cholesterol redistribution, and receptor-mediated lipid uptake, thereby shaping how macrophages handle extracellular lipid cargo and dying-cell-derived material in lipid-rich or metabolically stressed tissues (21). This cargo-processing function provides a mechanistic connection to lipid-associated macrophages and TREM2-positive macrophages, in which lipid stress, efferocytosis, and tissue remodeling are coupled to myeloid-state transitions (18, 19, 39, 40). The relevance of *APOE* to tumor-associated macrophage biology is further supported by pan-cancer single-cell studies showing recurrent *APOE*-expressing TAM programs across tumor types (41, 42). In HCC, the *APOE*-positive macrophage state described here may represent a tumor-associated manifestation of this lipid-adapted myeloid program, integrating lipid cargo handling, endolysosomal processing, redox adaptation, and macrophage-centered ligand–receptor communication. Together with the reduced tumor growth observed after host *Apoe* deficiency *in vivo*, these findings support a model linking *APOE*-associated macrophage lipid handling to a host microenvironmental program favorable to HCC growth. Cell-specific *Apoe* perturbation will be required to determine whether macrophage-derived *Apoe* is required for this *APOE*-associated macrophage-state program.

TREM2 provides a biologically plausible link between *APOE-*associated lipid handling and myeloid-state remodeling (39, 40). As a lipid-sensing receptor that signals through DAP12/TYROBP, TREM2 can translate lipid cargo recognition into macrophage functional programs (18, 40). In neurodegenerative disease models, the TREM2–APOE pathway has been shown to drive the transition of microglia from a homeostatic state toward a disease-associated phenotype, suggesting that ApoE can participate in myeloid-state regulation beyond its canonical role in lipid transport (21, 39). In the liver, *TREM2*-positive lipid-associated macrophages may support tissue repair and efferocytosis during metabolic injury, whereas recent HCC work showed that SQLE-high metabolism-archetype cancer cells promote oxLDL production, which engages TREM2 on TAMs and activates SYK–CEBPα signaling to induce protumor *TREM2*-positive TAM polarization (19, 20). This lipid-driven TAM program was linked to cancer-cell invasion, resistance to effector cytokines, CD8-positive T-cell dysfunction, and poorer immunotherapy response (20). Against this background, our observation that *APOE-*positive macrophages showed *APOE–TREM2* candidate communication together with lipid-processing and endolysosomal programs suggests that *APOE-*related lipid handling may cooperate with TREM2-linked lipid sensing to shape tumor-associated macrophage adaptation in HCC. Whether ApoE directly engages TREM2 in HCC macrophages, and whether this interaction is required for the observed macrophage state, will need to be tested experimentally (43).

These *in vivo* findings point to a host microenvironmental role for *Apoe* in the models tested. In the immunocompetent Hepa1–6 model, host *Apoe* deficiency was associated with smaller tumors, reduced vascularization, and increased CD8-positive cell infiltration. In NOG mice, *Apoe* knockdown in Hepa1–6 cells did not significantly change tumor volume or tumor weight, suggesting that the effect of *Apoe* may depend on immune or stromal context. In the *Nras-Myc*-driven liver tumor model, host *Apoe* deficiency was accompanied by reduced liver tumor burden and changes in myeloid programs, including lower lipid/cholesterol handling and lysosome/phagocytosis programs with higher inflammatory chemokine-associated activity. These findings connect the human *APOE-*positive macrophage signature with host-dependent microenvironmental remodeling associated with tumor growth, while leaving the precise host-cell source of *Apoe* to be defined experimentally.

Several limitations should be considered. First, the human single-cell findings were based on transcriptomic data. Additional spatial or protein-level validation in human HCC tissues will be needed to confirm the localization of *APOE-*positive macrophages and their relationships with the epithelial compartment, endothelial cells, fibroblast/CAF populations, and T/NK cells. Second, CellChat analysis can identify candidate ligand–receptor interactions but cannot establish direct ligand–receptor binding or downstream signaling (32). Third, although the *Nras-Myc*-driven liver tumor model complemented the subcutaneous Hepa1–6 model by providing a liver tumor setting, additional orthotopic and disease-background models will be useful to further evaluate how *Apoe-*related host effects operate across different HCC contexts. Fourth, systemic *Apoe* deficiency does not identify the exact host source responsible for the tumor phenotype. Because tumor-cell *Apoe* knockdown was tested in immunodeficient NOG mice, these data do not exclude a role for tumor-cell *Apoe* in an immunocompetent tumor microenvironment. Myeloid-specific or macrophage-specific *Apoe* perturbation, as well as tumor-cell *Apoe* perturbation in immunocompetent models, will be needed to test these contributions more directly. In addition, BODIPY 493/503 staining was performed *in vitro* using BMDMs and does not fully recapitulate the lipid milieu and multicellular interactions of the tumor microenvironment. Finally, *APOE* genotype and ApoE protein isoforms were not assessed in the patient samples, leaving open whether isoform-specific effects contribute to HCC immune-metabolic remodeling.

## Conclusions

Overall, this study identifies an *APOE*-associated lipid-handling macrophage state in HCC and links it to inferred ligand–receptor communication and host microenvironmental remodeling *in vivo*. Host *Apoe* loss attenuated tumor growth and was associated with altered myeloid programs, highlighting *APOE*-linked macrophage lipid handling as a potential entry point for understanding immune-metabolic remodeling and host-dependent microenvironmental changes in HCC.

## Data Availability

The human single-cell RNA-seq dataset analyzed in this study is available in the Genome Sequence Archive for Human (GSA-Human) under accession number HRA001748. This controlled-access dataset was accessed following approval under request number HREQ003429. The raw mouse single-cell RNA-seq data generated in this study are publicly available in the NCBI Sequence Read Archive (SRA) under BioProject accession number PRJNA1482280. Additional supporting information is provided in **Supplementary Tables S1–S8**.
